# Single-session dialectical behavior therapy skills training versus relaxation training for non-treatment-engaged suicidal adults: a randomized controlled trial

**DOI:** 10.1186/s40359-016-0117-4

**Published:** 2016-03-24

**Authors:** Erin F. Ward-Ciesielski, Connor B. Jones, Madeline D. Wielgus, Chelsey R. Wilks, Marsha M. Linehan

**Affiliations:** Behavioral Research & Therapy Clinics, University of Washington, 3935 University Way NE, Seattle, WA 98105 USA; Hofstra University, 135 Hofstra University, Hempstead, NY 11549 USA

**Keywords:** Dialectical behavior therapy, Treatment engagement, Treatment-seeking, Suicide, Randomized trial, Brief interventions

## Abstract

**Background:**

Individuals who are not engaged in treatment are commonly overlooked in the design of intervention trials targeting suicidal populations as a result of recruitment methodology that requires individuals to be referred from their current provider. In fact, research suggests that the majority of individuals who die by suicide have not been in contact with mental health services in the year before their death.

**Methods/design:**

A randomized controlled trial of two brief, one-session interventions for adults who are not engaged in mental health treatment. Inclusion criteria include 1) 18 years or older, 2) experiencing suicidal ideation in the past week, 3) have not received mental health treatment in the month prior to screening, 4) living within commuting distance to the research office, and 5) willing to consent to recording and assessment. Exclusion criteria are 1) non-English speaking and 2) significant cognitive impairment. Recruitment takes place in the community via flyers, radio, and online advertisements. Interested individuals are screened via telephone and those who are eligible attend a one-time in-person assessment and intervention appointment. During this appointment, they are randomized to a single-session intervention in which they are presented with either dialectical behavior therapy skills or supportive discussion and instruction in relaxation. Following the in-person appointment, participants complete three follow-up interviews via telephone at one-week, four-weeks, and twelve-weeks post-intervention. The primary outcomes are suicidal ideation, emotion dysregulation, and skills use. Secondary outcomes include depression, anxiety, self-efficacy, and treatment utilization. Exploratory outcomes are suicidal and intentionally self-injurious behaviors. Intent-to-treat analyses will be conducted on primary and secondary outcomes.

**Discussion:**

Suicidal individuals who are not engaged in mental health treatment are an understudied and significantly at-risk group for death by suicide. A better understanding of this population, targeted efforts to recruit and engage these individuals, and developing effective interventions for this group are critical areas for investigation in the field that this trial seeks to address.

**Trial registration:**

Clinicaltrials.gov identifier: NCT02236325; Date of registration: 05-Sept-2014

## Background

Suicide and suicidal behavior are major public health problems with enduring consequences. In 2013, suicide was the 10^th^ leading cause of death in the United States for all ages, and was among the top four causes of death for individuals aged 10 to 44 [1]. Worldwide, it is estimated that 800,000 individuals die by suicide each year [2]. In the United States (U.S.) alone, suicide claimed 41,149 lives in 2013 and costs an estimated $41.2 billion in medical and work-loss expenditures annually [1]. Furthermore, an estimated one million people report making a suicide attempt and more than two million people endorse suicidal thoughts each year in the U.S. [3]. Given the associated stigma with suicidal thoughts and behaviors [4], it is likely that these figures underestimate the scope of suicidal behavior.

While there is indication that some effective interventions for suicidal populations exist (e.g., dialectical behavior therapy [5]), there is a subpopulation that has been traditionally overlooked in this field: individuals who do not seek treatment in times of suicidal crisis. With only very rare exceptions, intervention studies targeting suicidal populations typically require that potential participants receive referrals into the trial from their current mental health providers. While a detailed review of the literature is outside the scope of this paper (see [6–8]), of the 56 studies that were reported in the above review papers, roughly 90 % have used medical or clinical referrals exclusively. This recruitment strategy presumes suicidal individuals who are already receiving mental health treatment comprise a sample that is representative of suicidal individuals as a whole, a point for which there is limited evidence. For example, a comprehensive review of 40 studies that reported rates of contact with mental health services prior to suicide found that in the year prior to death by suicide, only 32 % of individuals had made contact with mental health services, while 77 % of individuals had been in contact with primary care physicians. In the month prior to suicide, 19 % contacted mental health services and approximately 45 % contacted primary care [9]. Men and individuals aged 55 and older have also been shown to have the lowest rates of contact (i.e., 18 % of men and 11 % of adults 55 and older have been in contact with mental health services in the year prior to suicide [9]). These results call into question the assumption that treatment-engaged samples are generalizable to the wider suicidal population. In fact, the majority of individuals who die by suicide do not make contact with mental health services leading up to their death.

Another limiting factor of the existing literature is that many RCTs targeting suicidal samples rely on overly restrictive exclusionary criteria. Of the approximately 50 RCTs that have explicitly examined suicide as an outcome, more than a third excluded individuals at high risk of suicide. High risk of suicide being defined as in need of psychiatric treatment, in need of inpatient hospitalization, have been diagnosed with a mental disorder, and/or needing immediate treatment for suicidality [8]. More research to directly address the generalizability of the samples recruited in previously published trials is needed.

Fundamental to the traditional approach to recruiting participants for trials targeting suicidal populations is the assumption that suicidality is a symptom of a clinical disorder, which presupposes that suicidal individuals have a clinical diagnosis. However, Hamdi and colleagues [10] conducted a retrospective study of all suicides in a catchment area in the United Kingdom and found that those who were not in contact with mental health services in the year prior to their suicide death were less likely to have a mental health diagnosis or to report a history of previous self-harm. Additionally, Rhodes and colleagues [11] found higher rates of mental health contact for Canadian individuals experiencing depression and suicidal ideation (61.6 %) and depression alone (50.8 %) than for those experiencing suicidal ideation alone (26.8 %). Among Americans, an estimated 5.2 million adults who needed mental health treatment did not receive any mental health treatment in the past year [12]. Of adults who attempted suicide in the past year, only an estimated 67.2 % (752,000) received medical attention following the suicide attempt [12], indicating that there is a sizeable suicidal population that needs, but is not receiving mental health treatment, and thus not receiving a clinical diagnosis. This evidence suggests that relying on a documented diagnosis or the presence of symptoms that reach a diagnostic threshold may be misguided criteria to use in identifying and initiating intervention with individuals in need of mental health services.

Given the lack of targeted recruitment and outreach to individuals who are not already engaged in mental health treatment or in intervention research, the aim of this randomized controlled trial (RCT) is to evaluate the effectiveness of the brief, one-time, Dialectical Behavior Therapy Brief Suicide Intervention (DBT-BSI, [13]) for currently suicidal individuals who are not already engaged in mental health treatment. This intervention is compared to a relaxation intervention designed to control for non-specific factors.

## Methods/Design

In addition to evaluating the safety and feasibility of delivering this intervention to suicidal individuals who are not otherwise engaged in mental health treatment, this RCT’s aim is to estimate the immediate (one-week) and long-term (four- and 12-week) degree of change and variability of response to the DBT-BSI relative to a relaxation training (RT) control on the primary outcomes of suicidal ideation, emotion dysregulation, and skills use. Secondary outcomes of depression, anxiety, self-efficacy, and treatment utilization are also examined. Specifically, we hypothesize that, relative to the RT control condition:

- DBT-BSI will result in lower levels of suicidal ideation and emotion regulation;
- DBT-BSI will result in higher levels of skills use, in general, and in greater use of the specific skills taught in the DBT-BSI;
- DBT-BSI will result in lower levels of depression and anxiety; and
- DBT-BSI will result in greater utilization of mental health resources during the follow-up period.

### Participants

Participants were recruited from the community in the northwestern United States using radio, newspaper, community bulletin boards, and online advertisements. Advertisements ask individuals who are currently suicidal to contact the study researchers. This recruitment strategy has been successfully used in previous research targeting suicidal individuals [13]. Inclusion criteria include a) 18 years or older, b) experiencing suicidal ideation in the past week, c) having not received mental health treatment in the month prior to screening, d) living within commuting distance to the research office, and e) willing to consent to recording and assessment. Exclusion criteria are a) non-English speaking and b) significant cognitive impairment.

### Ethics and consent

All individuals recruited to the study underwent a process of informed consent during the initial phone screening assessment. Then, when participants arrived for their in-person appointment, they again underwent an informed consent procedure where they had a second opportunity to ask their study therapist questions before signing an agreement of informed consent. In addition to passing the National Institute of Mental Health ethics review, the study has been approved by the Human Subjects Division of the Institutional Review Board at the University of Washington. Any study modifications were approved by the University of Washington Institutional Review Board and, when appropriate, the National Institute of Mental Health.

Furthermore, a Data and Safety Monitoring Board (DSMB) was convened and a data and safety monitoring plan was developed to be sure that the study was conducted with the utmost conscientiousness. The Data and Safety Monitoring Plan indicated that the trial would be stopped if the majority of participants in either intervention condition exhibited significant and reliable worsening of suicidal ideation, suicidal, and/or self-injurious behavior. The DSMB met biannually to review trial progress and discuss any issues related to participants’ safety, adverse events, or the conduct of the trial.

### Confidentiality

Identifiable data (e.g., participants’ names, phone numbers) were maintained separately from their study data. Upon completion of data collection, identifiable information was destroyed. Non-identifiable data is coded for each participant using a unique, randomly-generated study identification number. This data is stored on a secure, encrypted server that is password-protected, to which only relevant study personnel have access. Additionally, a Certificate of Confidentiality was obtained from the National Institute of Mental Health to ensure an additional level of privacy protection for participants in the event of outside legal requests for identifying information.

### Procedure

Interested individuals who contacted the research office underwent an initial phone screening interview (T0) to determine study eligibility. Those who were eligible for the study were scheduled for on-site baseline assessment (T1) and intervention procedures. Following this in-person assessment and intervention appointment, participants completed three telephone follow-up interviews at 1- (T2), 4- (T3), and 12- (T4) weeks. Assessment domains include cognitive impairment (6-Item Cognitive Impairment Test) [14], suicidal ideation (Scale for Suicidal Ideation) [15] and suicidal behaviors (Lifetime Parasuicide Count) [16], emotion dysregulation (Difficulties in Emotion Regulation Scale) [17], depression (Patient Health Questionnaire Depression Module) [18], anxiety (Beck Anxiety Inventory) [19], skills use (DBT Ways of Coping Checklist) [20], and treatment utilization (Treatment History Interview; Linehan & Heard, unpublished work). Additionally, measures to prompt assessment and documentation of suicide risk were included (University of Washington Risk Assessment Protocol and the University of Washington Risk Assessment and Management Protocol) [21]. Participants were eligible to receive up to $45 in compensation for completing all study assessments (T1 = $5, T2 = $10, T3 = $10, T4 = $20).

The phone screening interview (T0) was conducted by the principal investigator and research assistants under the supervision of the principal investigator. The on-site baseline assessment (T1) and intervention appointments were conducted by three master’s-level graduate students in doctoral psychology programs. Weekly supervision and consultation were utilized for ongoing training and to monitor adherence to the intervention procedures.

### Randomization

A computerized minimization randomization algorithm was used to match participants on three variables that may confound analytic results. These three variables are identified gender, history of suicide attempts, and whether the participant was interested in mental health treatment. Participants were randomized using a 1:1 ratio, such that equal numbers of participants were assigned to each condition. Following completion of the T1 assessment, the therapist entered the participants’ data related to the three potentially confounding variables into the computerized program and participants were randomly allocated to one of the two conditions.

### Intervention conditions

The intervention was conducted on a one-on-one basis, was designed to last 45–60 min, and was completed in the same appointment and by the same therapist as the in-person assessment. After the T1 assessment was completed, participants were randomized into their treatment condition and the therapist used the appropriate intervention manual and materials related to the selected condition. At the start of each intervention session, participants are asked to briefly describe the factors they believed to be associated with their suicidal ideation and any patterns they had noticed in the occurrence of the ideation. At the end of each session, participants were provided a list of mental health resources.

### DBT-BSI

The DBT-BSI involved presenting participants with five pre-selected DBT skills [5, 22]:

1. Mindfulness (what to do with one’s attention/mind and how to engage in mindfulness practices);
2. Mindfulness of current emotions (observing and describing emotional experiences; labeling emotions; observing physical sensations over time);
3. Opposite-to-emotion action (blocking the behaviors prompted by emotions and instead acting opposite to or inconsistently with emotional urges);
4. Distraction (distracting attention by thinking about or doing something else); and
5. Changing your body chemistry (applying ice water to the face, engaging in intense exercise, pacing one’s breathing, and progressive muscle relaxation).

### RT

The RT procedures were designed to control for non-specific factors, such as the amount of time spent with a caring assessor, providing a rationale for usefulness of the information presented, and participant expectancies. The intervention was also based on principles of supportive therapy in which the assessor functions as a supportive, validating, and caring individual [23]. The intervention began with an open-ended discussion of the participant’s current life stressors and the ways they attempt to manage stress. Next, a rationale for relaxation was provided which emphasized the importance of building up resources to deal with stressors and difficult events more easily. Relaxation was not introduced as a skill. Instead, the therapist moved into encouraging the participant to try a relaxation practice and then walked the participant through a sensory awareness relaxation activity. The sensory awareness activity was based on a similar practice first developed by Goldfried and Davison [24] and involves the therapist reading a series of questions designed to prompt the participant to notice or pay attention to different sensations. Examples of the types of questions included in the practice are: “Can you feel your hair touching your head?” “Can you imagine something far away?” “Can you notice how one arm is warmer than the other?” The relaxation practice lasted approximately 10–15 min and was followed by a discussion of the impact of the practice on current stress and distress levels.

This type of activity provides confidence in the safety and potential effectiveness of the control condition; namely, there is reason to believe that relaxation techniques may partially serve a distress tolerance function and therefore was likely to provide immediate reduction in distress levels [25, 26]. However, if deficits in emotion regulation and distress tolerance skills are responsible for increased suicidal ideation (as would be predicted by Linehan’s theory [5, 27]), one would expect a greater reduction in suicidal ideation in the DBT-BSI condition over the RT control condition. Talking about skills, presenting relaxation practice as a skill, and problem-solving were all prohibited in the RT control.

### Follow-up

Participants were contacted by phone to schedule and complete their follow-up interviews (T2-T4). At the time of each interview, the next assessment was scheduled and then reminder calls were placed to confirm the appointment and reschedule as needed. Multiple attempts were made to reach each participant for each follow-up assessment to promote retention and to complete follow-up data collection. The follow-up interviews were conducted by research assistants under the supervision of the principal investigator (EW). All research assistants conducting follow-up interviews were kept blind to participants’ assigned intervention condition.

### Power calculation

A power analysis was conducted prior to the start of the study to provide a guide to sample size requirements. Calculations were based on feasible differences in treatment effect at the three-month follow-up assessment (T4). Based on Cohen’s [28] discussion of effect sizes, the effect size from the pilot study was consulted [13]. The effect size for the decrease in suicidal ideation observed during the pilot study was 0.56, which is considered a medium effect. In order to power the study with a medium effect size for detecting changes in suicidal ideation, the study will require 53 participants per intervention condition, using an independent sample *t*-test between the active and control condition (G*Power) [29].

### Analyses

Hierarchicial linear modeling [30, 31] will be used to assess differences between conditions over time. The within-subjects model (level-1), will include the estimates of the individual changes in repeated measures of suicide ideation, emotion dysregulation, skills use, depression, and anxiety assessed over time. The between-subjects model (level-2), will incorporate condition assignment as a predictor of the Level-1 growth parameters. Effect sizes will be computed using Feingold’s formula [32] and interpreted using Cohen’s guidelines [28].

Intervention effects for binary outcomes (i.e., use of the skills taught during the intervention, mental health treatment contact, and suicidal behaviors) will be evaluated in much the same way as continuous outcomes. Specifically, an extension of the generalized linear model, the generalized estimating equation (GEE) approach will be used [33, 34]. GEE is an increasingly popular approach to longitudinal and repeated measures designs, especially in the case of binary and categorical outcomes [35] owing to its simplicity relative to mixed models for fitting binary data. Effect sizes will be computed using relative risk ratios [36].

### Status of the trial

**Fig. 1:**
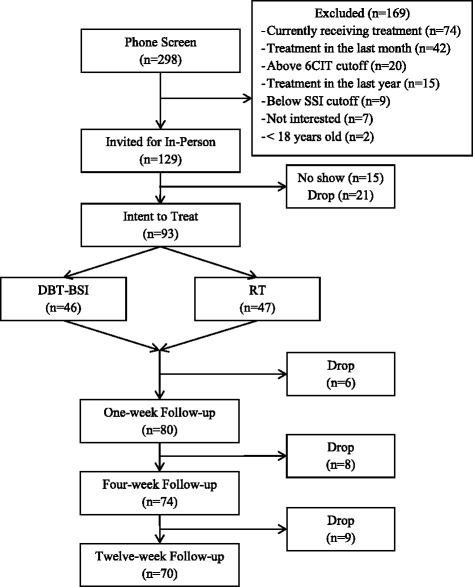
CONSORT Diagram

Recruitment to the study began January 2012 and ended December 2013. Final follow-up data was collected in March 2014 and data analysis is ongoing and expected to be completed by June 2016. The CONSORT diagram (Fig. 1) presents the flow of participants during recruitment, enrollment, and follow-up. Of note, 761 individuals contacted the research office to request additional information about the study. Of those requests, 463 individuals were subsequently not interested once the study was described or were unreachable after multiple attempts. In total, 298 individuals completed the phone screening assessment and 129 were determined to meet study inclusion/exclusion criteria and were invited for the in-person assessment and intervention appointment. Ninety-three individuals attended this in-person appointment and 70 were retained through the 12-week follow-up interview.

The total intent-to-treat sample size was 13 participants fewer than the pre-trial power analyses indicated would be necessary to detect a medium effect size change in suicidal ideation; however, based on power calculations for the full sample, this reduced sample will provide 80 % power to detect differences between the two groups in the medium range (Cohen’s d between 0.5 and 0.6 [28]) and less than adequate power to detect small differences. Additionally, the relatively high rate of retention will still allow us to draw meaningful conclusions from the primary and secondary outcomes of interest.

## Discussion

The importance of studying suicidal individuals who are not engaged in mental health treatment cannot be understated. Recruiting such individuals proved a challenge. Notably, the trial study inclusion criteria initially required that participants be without mental health treatment for one year prior to their enrollment; however, seven months into recruitment, it was determined that this requirement was slowing recruitment efforts and the criteria was modified to no mental health treatment one month prior to enrollment. Given the low rates of contact in the one month prior to suicide reported by Luoma and colleagues [9], we were confident that a non-treatment engaged population was still represented by this eligibility criteria modification. While this change suggests that more work is needed to maximize the effectiveness of outreach to this as-yet out-of-reach group, our success in reaching individuals who were not engaged in treatment suggests that at least some subset of this population is amenable to research and treatment participation. The impact of the results of this randomized controlled trial will provide future researchers with tools and strategies to enhance their own recruitment and outreach efforts as the field continues to develop and evaluate interventions for this high-risk group.

### Ethics approval and consent to participate

In addition to passing the National Institute of Mental Health ethics review (identification number: 1F31MH095257), the study has been approved by the Human Subjects Division of the Institutional Review Board at the University of Washington (application number: 40846). Consent was obtained from each participant prior to collecting any data from them.

### Consent for publication

Not applicable.

### Availability of data and materials

Complete data from this study can be obtained by contacting the first author once data analysis has been completed.

## Abbreviations

DBT: Dialectical behavior therapy
DBT-BSI: Dialectical behavior therapy brief suicide intervention
DSMB: Data and Safety Monitoring Board
RT: Relaxation training
